# A critical review of American Trypanosomiasis (AT) in dogs with the current distribution of cases in Brazil and a diagnostic approach for veterinary clinicians

**DOI:** 10.1007/s11259-025-11047-6

**Published:** 2026-01-24

**Authors:** José Atanásio de Oliveira Neto, Geovana Mergulhão da Silva, Samuel Souza Silva, Tatiene Rossana Móta Silva, Gílcia Aparecida de Carvalho, Leucio Câmara Alves, Rafael Antonio Nascimento Ramos

**Affiliations:** 1 Laboratory of Parasitology, Federal University of the Agreste of Pernambuco, Garanhuns, 55292-278 Brazil; 2 Graduate Program in Animal Bioscience, Federal Rural University of Pernambuco, Recife, 52171-900 Brazil; 3 Department of Veterinary Medicine, Federal Rural University of Pernambuco, Recife, 52171-900 Brazil

**Keywords:** Canine, One health, Epidemiology, American trypanosomiasis, Trypanosoma cruzi

## Abstract

*Trypanosoma cruzi* (Kinetoplastea: Trypanosomatidae) is the etiological agent of American Trypanosomiasis (AT). Although knowledge of natural infection by *T. cruzi* in dogs has increased worldwide relatively few studies have evaluated this occurrence in Brazil, with much of the available information restricted to isolated case reports. The aim of this research was to provides a critical review of AT in dogs demonstrating the current distribution of cases in Brazil and to propose a diagnostic approach for veterinary clinicians. This review comprised a comprehensive search of scientific articles and case reports published, available at PubMed, Scielo and Google Scholar websites. A combination of keywords including “American Trypanosomiasis”, “Chagas disease”, “dogs”, “*T. cruzi*”, and “Brazil” was used for the search. Data from this review underscores the importance of dogs as domestic hosts of *T. cruzi* and provides the current distribution of this infection in these animals in Brazil. Additionally, it reveals important knowledge gaps regarding veterinarian awareness of *T. cruzi* and its diagnostic methods in small animal clinics. The diagnostic flowchart presented here serves to guide veterinarians to perform a proper diagnostic using currently available methods. The prevention of AT in dogs faces several challenges, including the domiciliation of triatomines and the misdiagnosis of cases. These factors contribute to the ongoing presence of untreated animals, which can serve as important sources of infection for triatomine vectors and ultimate risk of human infections. For the AT control, it is important to reinforce the necessity of a One Health approach and to underscore the role of veterinarians in this process. It is pivotal to focusing on diagnosing and treating humans and dogs, controlling vectors, and improving human dwellings, especially in rural areas. Finally, cases of AT in dogs in Brazil distribute heterogeneously in all five regions of the country, with a slight predominance in the Northeast region.

**Supplementary Information:**

The online version contains supplementary material available at 10.1007/s11259-025-11047-6.

## Introduction

*Trypanosoma cruzi* (Kinetoplastea: Trypanosomatidae) is a protozoon of great importance in public health, transmitted by triatomine vectors, and considered the etiological agent of American Trypanosomiasis (AT) (WHO, 2015). AT is endemic in 21 countries in the Americas, and currently, approximately 75 million people live in risk areas of infection (WHO, 2024). It is estimated that from 6 to 8 million humans are infected, and more than 10,000 human deaths occur every year (WHO, 2021). *T. cruzi* infects more than 100 species of mammals, including rodents, marsupials, armadillos, bats, carnivores and primates (Zingales et al. 2012; Zingales and Bartholomeu 2022). Dogs are considered important domestic hosts and may act as sentinels for human infections (Montenegro et al. 2002; Luciano et al. 2009). The knowledge of natural infection by *T. cruzi* in pet animals has increased over the last years in the Americas (Araújo-Neto et al. 2019; Zecca et al. 2020; Durães-Oliveira et al. 2024). Previous studies have demonstrated the presence of infected dogs in the United States (Shadomy et al. 2004; Tenney et al. 2014; Allen and Lineberry 2022), Mexico (Estrada-Franco et al. 2006; Avalos-Borges et al. 2024), Colombia (Jaimes-Dueñez et al. 2020; Cantillo-Barraza et al. 2021), Venezuela (Crisante et al. 2006; Berrizbeitia et al. 2013; Diaz-Bello et al. 2016; Gutiérrez et al. 2023), Argentina (Gürtler and Cardinal 2015; Monje-Rumi et al. 2015), Brazil (Araújo-Neto et al. 2019; Cruz et al. 2020; Costa et al. 2021; Santos et al. 2022) and Panama (Saldaña et al. 2015).

The pivotal role of dogs in the epidemiology of AT has increasingly captured the attention of the scientific community. Dogs stand out as important domestic hosts that potentially influence cases of AT in humans (Gürtler and Cardinal 2015). These animals have been considered the only host species capable of developing clinical forms similar to those observed in human patients (Barr et al. 1991; Gürtler et al. 2007). In dogs, *T. cruzi* infection can occur through different transmission routes. Despite the classical vector-mediated route of transmission, the oral route is considered more epidemiologically important for these animals. The infection usually occurs through the ingestion of infected triatomines during predatory behavior, or through the consumption of raw tissues from parasitemic wild reservoirs. Such exposures usually result in the introduction of a high parasitic burden, which may influence the severity of the acute phase and the course of the infection. Stray dogs that frequently come into contact with vectors and wild animals, further increase the likelihood of oral transmission (Cleaveland et al. 2006; Pavarini et al. 2009; Durães-Oliveira et al. 2024).

In Brazil, few studies evaluate the natural infection by *T. cruzi* in dogs with most limited to individual case reports rather than comprehensive investigations (Costa et al. 2021). Despite the scarcity of data, some serological surveys have indicated a seroprevalence ranging from 0.7% in the state of Bahia (Leça Junior et al. 2013) to 53% in the state of Mato Grosso do Sul (Porfirio et al. 2018). Clinically, the disease features an acute phase, in which animals may develop intense myocarditis with marked parasitism of myocytes and necrotic lesions of non-parasitized cardiac cells (Andrade et al. 1980; Barr, 2009), followed by the chronic phase, in which animals may vary from asymptomatic to presenting myocardial failure and ventricular arrhythmias (Barr, 1992). The lack of studies focusing on the natural occurrence of AT in dogs impairs a reliable understanding of the natural history of this disease in the canine population from Brazil. Most of the knowledge accumulated in the last decades comes from experimental infections or post-mortem studies (Montenegro et al. 2002; Camacho and Alves, 2007).

Urgent collaborative efforts are needed to gather epidemiological data on *T. cruzi* occurrence in dogs across Brazil, which will help map distribution areas and establish diagnostic guidelines for veterinary clinicians. The aim of this research was to provides a critical review of AT in dogs, demonstrating the current distribution of cases in Brazil, and to propose a practical diagnostic approach for veterinary clinicians.

## Materials and methods

While AT in dogs has been few reported and studied in Brazil and data are scant, a critical review involving a comprehensive search of scientific articles and case reports published in online databases such as PubMed (USA National Library of Medicine National Institutes of Health/National Center for Biotechnology Information Search database), Scielo (Scientific Electronic Library Online) and Google Scholar was written.

Keywords including “American Trypanosomiasis”, “Chagas disease”, “dogs”, “*T. cruzi*”, and “Brazil” were combined to search for articles. Inclusion criteria were (1) articles indexed in the previously cited databases, (2) original studies in Portuguese or English, and (3) published between 1969 and 2024. The search excluded secondary publications such as books, monographs, dissertations and theses. For the geographical distribution of cases, it was considered only studies on natural infection.

After an initial analysis of the titles, sixty-two peer-reviewed articles related to *T. cruzi* infection in dogs in Brazil were selected. Twenty-eight articles were excluded, as they were literature reviews or experimental studies, and all remaining 34 articles were used for the determination of the distribution of cases. Additional references published elsewhere were considered for the written of biological and epidemiological aspects of AT.

This critical review presents a limitation related to the risks of selection and the possibility of biases that accompany non-systematic syntheses. To mitigate this limitation, it was utilized all indexed research articles, written in Portuguese or English and published between 1969 and 2024, excluding books, monographs, dissertations and theses.

### The etiological agent: *Trypanosoma cruzi*

*Trypanosoma cruzi* is the causative agent of AT, a protozoon that may infect several animal species (Zingales et al. 2012; Dias et al. 2016). Currently, *T. cruzi* lineages are differentiated by highly heterogeneous populations into distinct genetic groups called Discrete Typing Units (DTUs): TcI, TcII, TcIII, TcIV, TcV, TcVI and TcBat (Zingales et al. 2012; Marcili et al. 2009). The genotype TcI is widely distributed throughout the Americas and has been detected in wild hosts from several mammalian Orders including Artiodactyla, Carnivora, Chiroptera, Cingulata, Didelphimorphia, Pilosa, Primates, Rodentia, and Xenarthra (Magalhães et al. 2022).

DTUs are currently associated with different geographic distributions and ecological niches, as well as virulence, pathogenicity and tissue tropism in the host. These features have implications for understanding the ecoepidemiology of *T. cruzi* and the development of measures of disease control and surveillance strategies (del Puerto et al. 2010; Miles et al. 2009). Most DTUs can cause human infections (Miles et al. 2009; Zingales et al. 2012; Zingales and Bartholomeu 2022), but in dogs, DTUs TcI and TcIV are more commonly reported (Ramírez et al. 2013; Dumonteil et al. 2020). The TcI has been the main DTU identified in vectors in the American continent (Brèniere et al. 2016). A study conducted in Venezuela demonstrated that TcI predominated over TcII, but with both of them being detected in wild, peridomestic and domestic sources including triatomine-bugs (Añez et al. 2009). Reports of TcBat in humans are restricted to the detection of this DTU in a mummy in Chile and in a child in Colombia (Guhl et al. 2014; Ramírez et al. 2014). In Brazil, TcI has also been isolated from dogs in the state of Ceará (Bezerra et al. 2014).

### The transmission of *T*. *cruzi* to dogs

*T. cruzi* is a parasite primarily transmitted by vectors, which presents a complex life cycle associated with a wide range of hematophagous triatomines (de La Fuente et al. 2007; Lima Neiva et al. 2021). Briefly, triatomine feeds on an infected host and ingests trypomastigote forms of the parasite. In the insect’s midgut, they differentiate into epimastigotes, and after migration to the rectal ampulla, they differentiate into metacyclic trypomastigotes in a process known as metacyclogenesis. Metacyclic trypomastigotes are the infective stage for vertebrate hosts and are eliminated in feces during the blood meal, penetrating a new susceptible host (Teixeira et al. 2009; Moretti et al. 2020).

The predominant transmission route in domestic dogs remains unclear (Durães-Oliveira et al. 2024). Although oral transmission through the ingestion of infected vectors is common, the consumption of contaminated mammalian meat, or, less commonly, via lactogenic transmission from mother to puppies represents other alternative routes of transmission (Barr et al. 1995; Barr 2009). In areas close to forests, the high prevalence of AT in dogs can be attributed to their increased exposure to the wild environment, which enhances the opportunities for contact with *T. cruzi* wild reservoirs and respective vectors. This proximity facilitates direct and indirect interactions between dogs and wildlife species, because human dwellings are often located very close to, or sometimes within, forested areas (Xavier et al. 2012).

In wild carnivores, the ingestion of infected vectors and vertebrate hosts is a primary mechanism for acquiring *T. cruzi* (Jansen et al. 2018). Despite the lack of scientific evidence, this transmission route is likely possible for domestic dogs (Rokhsar et al. 2023). While insecticide-based control strategies are widely used to reduce the transmission of *T. cruzi* by targeting triatomine vectors, recent studies indicate that such interventions can have unintended consequences for canine health, particularly in regions of low endemicity (Rokhsar et al. 2023).

Since 1928, the vertical transmission of *T. cruzi* in dogs has been known. In this first report, the parasite was detected in various organs of newborn puppies from bitches with acute and chronic infections. Notably, the observation that not all puppies in the same litter were infected suggests complex dynamics affecting transmission efficiency (Souza Campos 1928). This vertical transmission through congenital or lactogenic passage has important epidemiological implications because even with vector control, new parasite reservoirs may constantly emerge, complicating efforts to control *T. cruzi* infection.

Although congenital transmission is a possible route of infection, results from experimental studies using animal models, such as rats and mice, have been contradictory. Vertical transmission most likely occurs primarily during the acute phase of maternal infection when parasitemia is highest. Moreover, the presence of the parasite in breast milk has not been consistently demonstrated, raising questions about the role of nursing in transmitting *T. cruzi* to offspring (Moreno et al. 2003). In a previous study, seronegative mothers produced litters with both seropositive and seronegative puppies, supporting the hypothesis of vectorial transmission (Curtis-Robles et al. 2017). More recently, fetuses from bitches with detectable parasite DNA in cardiac tissue exhibited the highest parasitic loads in both blood and myocardial tissue, suggesting a possible correlation between maternal parasite localization and the severity of fetal infection (Avalos-Borges et al. 2023). Although the mechanisms underlying this route remain insufficiently explored, transmission may occur during partial or complete placental detachment. Additionally, placentas that appear clinically normal may harbor microscopic or structural abnormalities that facilitate fetal infection (Avalos-Borges et al. 2023).

#### The role of dogs as reservoirs

The role of domestic dogs as a reservoir of *T. cruzi* has been debated for a long time, and currently, they are considered an important domestic reservoir due to their proximity to humans (Crisante et al. 2006; Estrada-Franco et al. 2006; Ramírez et al. 2013). Dogs are highly susceptible to *T. cruzi*, likely due to an insufficient innate immune response to control the initial parasitemia during acute infection, (Travi 2019; Jaimes-Dueñez et al. 2020). As a result, high parasitemia in the acute phase occurs (Gürtler et al. 2007; Gürtler and Cardinal 2015). While the infection progresses to the chronic phase, the parasitemia decreases, and the ability of the triatomine to be infected reduces. At this stage, the parasites are concentrated in specific tissues (e.g., cardiac muscle), reducing their presence in the bloodstream. Thus, even if triatomines continue to feed on a chronically infected host, the likelihood of acquiring the parasite becomes significantly low (Araújo et al. 2002).

The territorial behavior of some dogs, combined with triatomine around human dwellings, increases the risk of dog infection by *T. cruzi* (Elmayán et al. 2019). Dogs kept in dwellings with earthen or wooden walls showed higher seroprevalence for *T. cruzi* compared to those housed in masonry homes. This finding suggests that buildings constructed with rustic materials favor triatomine infestation due to the presence of cracks and structural irregularities that provide shelter for vectors. The increased abundance of these insects in such environments elevates the risk of canine exposure, either through infective bites or accidental vector ingestion (Jaimes-Dueñez et al. 2020). Analysis of blood sources in triatomine populations in households shows that more than 50% of these insects feed on non-human hosts (Catalá et al. 2004), highlighting the importance of domestic animals as blood sources.

A relevant study documented a rare case of human infection by *T. cruzi* associated with direct contact with the blood of an infected dog residing in an endemic area of Venezuela, where approximately 70% of households were infested by triatomines. In this case, four children were infected after exposure to the blood of a wound from an infected dog. The direct infection route via bodily fluids, despite of considered uncommon, it might occur in endemic regions (Crisante et al. 2006; Añez et al. 2009).

The role of these animals as reservoirs goes beyond their proximity to human beings and high parasitemia; they may serve as a link between the domestic and sylvatic transmission cycles (Ramírez et al. 2013). Dog exposure patterns and the trophic relationship of these animals with vectors determine a constant entry of *T. cruzi* into the domestic transmission cycle, which qualifies dogs as amplifying hosts of *T. cruzi* in endemic areas (Gürtler et al. 1986). The animal movement among cities and from rural to urban areas can amplify the transmission dynamics of *T. cruzi*, creating new epidemiological scenarios in urban areas where transmission is usually rare or absent. These events highlight the critical role of these animals as potential reservoirs for the parasite (Murcia-Cueto et al. 2024). It is pivotal for pet owners to keep their animals with restricted access to areas potentially infested by triatomines in their homes or neighborhoods.

### The clinical presentation of *T*. *cruzi* infection in dogs

To our knowledge, dogs have been considered the only animal that develop clinical signs similar to those observed in humans (Barr et al. 1991, 1995). For this reason, they have been widely studied as a biological model, especially for cardiac manifestations (Tafuri et al. 1988).

During the acute phase of infection, *T. cruzi* trypomastigotes circulate within the bloodstream and can enter macrophages, disseminating throughout most body tissues (Freitas et al. 2022). At this time, *T. cruzi* causes an intense myocarditis by the penetration into cardiomyocytes (Barr, 1992). In this phase, animals may be asymptomatic or develop clinical signs disease rapidly. In experimentally infected young animals, acute myocarditis begins in the atrium and spreads through the interventricular septum toward the ventricles (Andrade et al. 1980). Generally, the lesion is localized in the right atrium and the free wall of the right ventricle. Heart block occurs in the terminal stage and is associated with severe inflammation. Significant electrocardiogram (ECG) changes are also observed in experimental infected animals, reflecting atrial involvement (Andrade et al. 1980).

Adult dogs diagnosed with AT may survive longer than puppies (Kjos et al. 2008; Barr 2009). The acute phase occurs from the first few days after exposure up to approximately two and a half months. The infected dog presents high parasitemia, low IgG antibody titers, and may exhibit nonspecific clinical signs such as muscle pain, arthralgia, vomiting, prostration, and monoclonal phagocytic involvement, including splenomegaly, hepatomegaly and lymphadenopathy (Matthews et al. 2021). In puppies, generalized lymphadenopathy, pale mucous, splenomegaly, and hepatomegaly have been observed. Some studies suggest that dogs infected after 6 months of age do not show signs of acute disease and enter the chronic phase approximately 30 days after infection (Barr 2009).

Similar to humans, infected dogs that survive the acute phase also enter the chronic phase, either asymptomatic or symptomatic (Barr 2009; Matthews et al. 2021). In symptomatic cases, this phase is featured by myocardial failure and ventricular arrhythmias (Barr et al. 1995). In a study conducted by Kjos et al. (2008) in the state of Texas, United States, cardiac enlargement was reported as the most common clinical sign (33.6%), followed by lethargy (28.7%), anorexia (23%), ascites (22.1%), and other cardiac manifestations (21.3%). Important chronic lesions include fibrosis and cardiomyocyte necrosis, likely due to the inflammatory processes (Meyers et al. 2020). Right-sided heart failure, and eventually left-sided heart failure, can also be observed, leading to pulse deficits, ascites, pleural effusion, hepatomegaly, and jugular venous congestion (Barr 2009; Stoner and Saunders 2020; Matthews et al. 2021).

In addition to all cardiac manifestations, megaesophagus and megacolon can also be observed in dogs (Nogueira-Paiva et al. 2015). *T. cruzi* triggers an inflammatory reaction in the esophagus and colon, causing myenteric denervation. In experimentally infected dogs, ganglions and periganglionitis of the Auerbach plexus have also been observed, leading to significant neuronal lesions (Bahia et al. 2002). The survival rate and prognosis can be unpredictable in untreated chronically infected dogs.

### *Trypanosoma cruzi* diagnoses in dogs

**Fig. 1:**
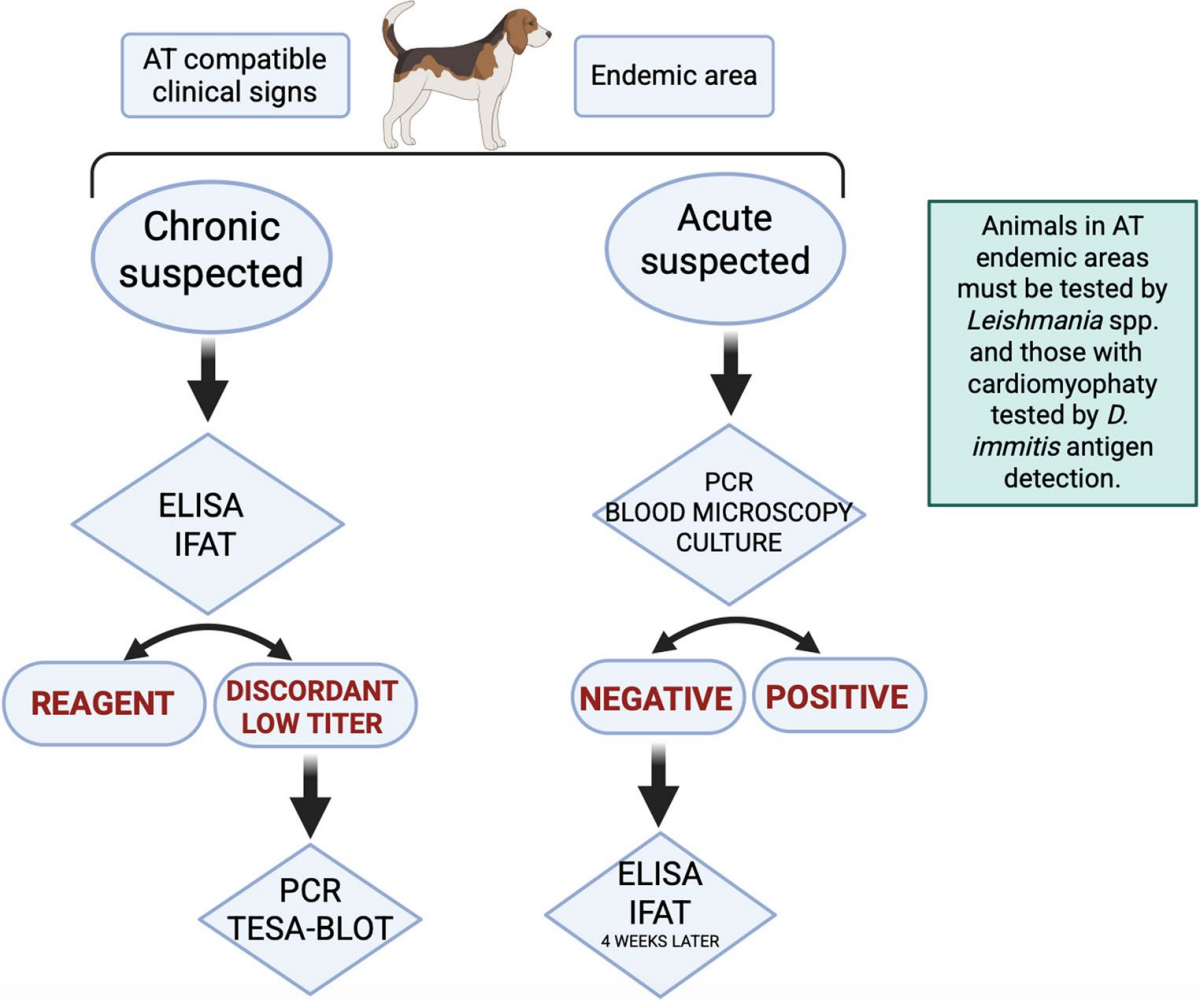
Diagnostic flowchart to guide veterinary clinicians on the AT diagnosis in dogs

Diagnosing *T. cruzi* infection in dogs is challenging and complex and should be carried out through an integrative approach involving epidemiological data, clinical signs, and laboratory examinations (Santana et al. 2012; Freitas et al. 2022). In the Table 1 we summarized the main diagnostic tools available for *T. cruzi* infection in dogs in Brazil. Additionally, a diagnostic flowchart has been proposed in the Fig. 1.

**Table 1:** More common methods of diagnosis available for detection of *T. cruzi* infection in dogs in Brazil according to the infection stage

	Method of diagnosis (biological sample)	Acute infection	Chronic infection	Main features
Parasitological	Microscopy (blood)	Yes	Yes^1^	Parasitemia depends on the stage of infection. Direct parasite detection is mainly applicable during the acute phase of the infection. During the chronic phase, parasitemia tends to be low and intermittent, reducing the sensitivity.
	Culture (blood)	Yes	Yes	
Immunological	ELISA (serum/plasma)	Yes^2^	Yes	Sensitivity varies among techniques and according to the genetic variability of *T. cruzi*. There is the possibility of cross-reaction with other trypanosomatids, such as *Leishmania* spp., in an area of endemic overlap. In the acute phase, the sensitivity is low.
	IFAT (serum/plasma)	Yes^2^	Yes	
	TESA-BLOT^3^ (serum/plasma)	Yes	Yes	High sensitivity and specificity. No cross-reactivity with other trypanosomatids.
Molecular	PCR and derivations (blood)	Yes	Yes	Parasitemia depends on the stage of infection. DNA detection is mainly applicable during the acute phase of the infection. During the chronic phase, parasitemia tends to be low and intermittent, reducing the sensitivity.

^1^ Limited sensitivity due to the low and intermittent parasitemia

^2^ Consider the detection period of antibodies of about 4 weeks post-infection

^3^ Despite not being a standard method used for diagnosis in Brazil, studies have proven its importance for diagnosis in endemic areas for other trypanosomatids

Parasitological methods, such as microscopy of blood samples, are valuable for confirming *T. cruzi* infection in the acute phase, up to 2.5 months post-infection, when parasitemia is higher (Jansen et al. 2008). This type of diagnosis presents high sensitivity and specificity during the acute phase (Vitt et al. 2016; Pereira et al. 2021).

Serological techniques for detecting antibodies against *T. cruzi* may also be helpful for diagnosis (Montenegro et al. 2002; Barr 2009; Matthews et al. 2021). These methods include enzyme-linked immunosorbent assays (ELISA) or immunofluorescence antibody tests (IFAT). Overall, the sensitivity and specificity of serological tests are higher than 90% and increase throughout the infection (Freitas et al. 2022). It is important to note that a reagent serology does not always indicate infection, but means exposure (Barr 2009). The sensitivity varies across different serological tests and the specificity of these tests can also be affected by cross-reactivity with other trypanosomatids, such as *Leishmania* spp. (Meyers et al. 2021; Freitas et al. 2022). The cross-reactivity occurs because these parasites are phylogenetic closely related, and a positive result in a leishmaniasis endemic area might be carefully interpreted (Bracho-Mora et al. 2015; Costa et al. 2021).

Some techniques (e.g., rapid diagnostic test and TESA (Trypomastigote excreted-secreted antigen-Blot) that were proposed to diagnose *T. cruzi* infection in humans, it has been adapted for dogs (Meyers et al. 2019; McClean et al. 2020). In veterinary medicine, the use of serological tests for *T. cruzi* infection remains limited by the unavailability of specific test kits for this species, with few laboratories offering options for dogs (Busselman and Hamer 2022). In 2020, the diagnostic performance of a rapid test based on the small trypomastigote surface antigen (TSSA) (Chagas Sero K-SeT) was evaluated. However, the low sensitivity presented by the test indicated the need of further assessments to improve the its performance (McClean et al. 2020). Recently, a lateral flow immunochromatographic rapid test (Chagas-LFRT) was adapted for dogs and its use has been proposed as a safer, reliable and low-cost diagnostic tool for surveillance (Rodrigues et al. 2022).

In the chronic phase, parasitemia in dogs is low and intermittent, reducing the sensitivity of parasitological methods. However, the production of anti-*T. cruzi* antibodies reach detectable levels and can be identified through indirect immunoassays. IgM in dogs declines sharply about three months after infection. In comparison, IgG increases until 15 months, gradually decreasing until two years, after which it appears to stabilize throughout the infection (Lana et al. 1991).

More recently, molecular methods, such as PCR and derivations, have been used to detect *T. cruzi* DNA from various biological samples. This technique confirms the infection through detection of genomic material of the parasite. Molecular amplification tests, such as PCR, are more sensitive and capable of identifying the parasite’s genetic strain. However, they are costly and require well-equipped laboratories with highly trained personnel, limiting their use in areas without adequate infrastructure (Pereira et al. 2021). Moreover, the effectiveness of PCR in epidemiological surveys is limited, making the appropriate selection of primers and DNA targets essential to ensure sensitivity and specificity across different geographic regions and parasite DTUs (Pinazo et al. 2023).

Molecular techniques provide detailed information about the genetic strain of *T. cruzi* and allows differentiation of DTUs, making it a valuable tool for research. In dogs, qPCR (quantitative PCR) uses the parasite’s kDNA minicircles as a marker, offering a rapid and highly sensitive diagnostic option (Durães-Oliveira et al. 2024). Although still less common, the development of new molecular amplification-based diagnostic approaches has advanced for resource-limited regions. One example is loop-mediated isothermal amplification (LAMP), which shows potential for efficiently detecting *T. cruzi* infections, especially in endemic regions (Pinazo et al. 2023; Durães-Oliveira et al. 2024).

Complementary exams such as ECG and echocardiography have been recommended for screening cardiac diseases (Barr 2009; Montenegro et al. 2002; Meyers et al. 2021; Avalos-Borges et al. 2024). Serum cardiac troponin I (cTnI) has been increasingly studied as an early biomarker of parasitic myocarditis in dogs with AT (Matthews et al. 2021; Meyers et al. 2021). Although these tests do not confirm the *T. cruzi* infection, they may help support the laboratory diagnosis (Montenegro et al. 2002; Matthews et al. 2021; Avalos-Borges et al. 2024). The main limitation of these exams lies in their inability to confirm the presence of the parasite, as well as the challenges in detecting the disease in its early stages or when cardiac manifestations are mild.

Unfortunately, the routine diagnosis of canine AT faces several challenges, and despite its importance for public and veterinary health, there are few standardized diagnostic options available for animals in the veterinary clinic of companion animals (Meyers et al. 2017; Freitas et al. 2022; Zelachowsk et al. 2024). Additionally, clinical information about animals is often less robust or inaccessible, mainly due to difficulties in data collection. Any intervention that aims to reduce infection in dogs may reduce the risk of the disease in humans; therefore, the veterinary diagnosis of AT should not be overlooked (Busselman et al. 2025). The development of new diagnostic markers able to detected early infections and animals chronically infected are encouraged.

Any test employed for AT diagnosis should be used based on the suspicious phase of infection and interpreted on the light of a comprehensive scenario that considers both clinical and epidemiological factors. All non-direct diagnostic techniques (e.g., serology) that are available may be used. However, results should be carefully interpreted, especially in endemic areas for canine leishmaniasis, where the possibility of cross-reactivity exists.

### The geographic distribution of AT in dogs in Brazil

**Fig. 2:**
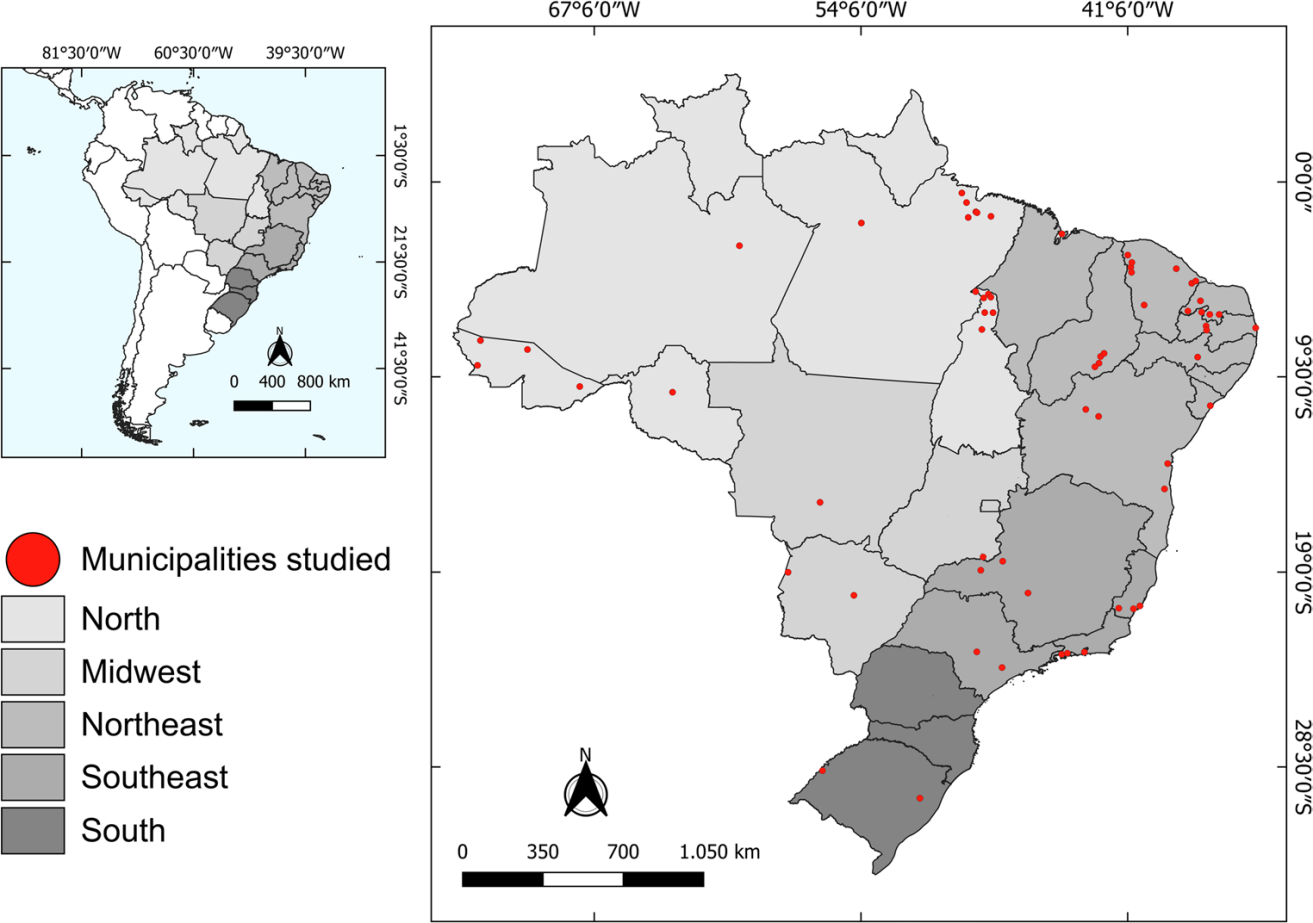
Distribution of natural *T. cruzi* infection in dogs in Brazil. Legend: Data sources of this figure were peer-reviewed reports summarized in the Supplementary file 1. A kernel density map was not used because geo-coordinates were unavailable. Only municipalities with positive AT cases have been represented in the figure

In Brazil, most studies on *T. cruzi* involve experimental infections since the disease in dogs resembles that of humans (Andrade et al. 1980; Caliari et al. 2002; Carvalho et al. 2019). The few reports on natural *T. cruzi* infection, especially in endemic areas, impair a reliable analysis of the true prevalence and distribution of canine cases across the country (Fig. 2; Supplementary file 1).

Although scarce, studies have demonstrated high seroprevalence in endemic areas. For example, seroprevalences ranging from 22% (*n* = 96) to 71% (*n* = 649) have been observed in the state of Ceará (Bezerra et al. 2014; Xavier et al. 2012), from 11% (*n* = 52) to 71% (*n* = 649) in the state of Piauí (Xavier et al. 2012; Herrera et al. 2005), 42.5% (*n* = 40) in Rio Grande do Norte (Araújo-Neto et al. 2019) and 10% (*n* = 168) in Sergipe (Cruz et al. 2020). These data underscore the high exposure of dogs to *T. cruzi* infections in endemic regions, particularly in the semiarid northeastern region of the country. On the other hand, a more recent study revealed a seroprevalence of only 0.5% (*n* = 392) in an area characterized by the Atlantic Forest biome in Bahia (Souza et al. 2018).

These discrepancies can be attributed to several factors, such as the population density of triatomines, the specific socio-environmental conditions of each region, and the level of dogs’ exposure to *T. cruzi*. These variations highlight the complexity of the epidemiological dynamics of the infection in endemic areas and the need for a fully understanding of the factors influencing its distribution.

The high prevalence rate in dogs is important in the context of the circulation of *T. cruzi*, even after public policies to control and reduce triatomines in households. In the semiarid region of Northeastern Brazil, raising animals in peridomestic environments close to human dwellings is cultural and important to ensure assets and family (Bezerra et al. 2014). Additionally, in this region there is a cultural hunting behavior by the population, which may expose dogs to contaminated meat of wild animals increasing the risk of infection. Despite speculative, this hypothesis needs to be further assessed.

The domiciliation of triatomines, combined with the high prevalence of *T. cruzi* among dogs in some regions, strengthens the relationship between the enzootic cycle and human populations. The availability of domestic hosts in homes can directly affect the choice of food source by the triatomine, reducing the rates of human-vector contact and the transmission of the parasite (Freitas et al. 2018).

Changes in the natural landscape are important for the AT epidemiology because mammals with generalist feeding behavior such as *Didelphis* spp. are favored over those specialist ones. The generalist feeding behavior is characterized by the ability to feed from different sources of meals, which favor the increasing and spreading of these animals’ population in a given area. This leads to an increase in the infection rate in generalist mammals that may be suitable hosts for *T. cruzi*. This fact contributes to the increase in the amplification and circulation of the parasite within an area, favoring vector infection and exposing more dogs to *T. cruzi* (Xavier et al. 2012).

AT in human patients in Brazil has been treated in light of a governmental policy since 1975, and the distribution of cases is well-documented, despite the limitations of public notification databases. Data on the occurrence and distribution of AT in dogs are scarce, and prospective, geographically resolved studies are welcomed. The geographical presentation of cases represented in the present study (see Fig. 2) is limited because most of the research used as a data source did not present geocoded data, impairing a more accurate representation of the distribution of cases across the country.

## Final considerations and future perspectives

Data from this review underscores the importance of dogs as domestic hosts of *T. cruzi*, provides the current distribution of this infection in Brazil based on published data, and propose a diagnostic approach for veterinary clinicians. Despite of all information gathered; it is evident that the knowledge on the natural occurrence of AT in dogs in Brazil is limited. Most veterinarians in this country are not completely aware about the risk of dog infection and clinical signs presented by infected animals. While the majority of cases of dogs with cardiomyopathy are attributed to heartworm disease, the suspicion of AT remains neglected. Educational activities with health professionals and local community should be stimulated to increase the knowledge of population about *T. cruzi* infection in dogs and all risk factors involved in the occurrence of AT in animals and humans.

Commercial diagnostic tests are not currently available, and the majority of cases reported are based on research studies, post-mortem confirmation, or even occasional findings. It is also important to note that in Brazil, endemic AT areas predominate in regions of social vulnerability where animals are not regularly submitted to veterinary care, making it difficult to diagnose infection. Public health policies for AT control should include also the monitoring of dogs in endemic areas and to promote continuing educational programs for health professionals. The increase of the knowledge of AT in dogs and the inclusion of these information in public databases are pivotal to establish focused preventive measures for human populations living in critical endemic areas.

The prevention of *T. cruzi* infection in animal and human populations involves several challenges, including the domiciliation of triatomines, the infection in domestic and wild animals, the difficulty of dog diagnosing, the free movement of dogs from endemic to non-endemic areas through animal trade and adoptions, and the cultural behavior of some populations that favor the exposure of dogs to vectors and contaminated carcasses during hunting activities. All these factors reinforce the necessity for an One Health approach that integrates the health of animals, humans, and the environment. Community engagement combined with public health policies is essential to keeping infection rates low and preventing the emergence of AT in animals and humans. We recommend updating public policies used for AT control with the insertion and valorization of veterinarian professionals in the whole prevention process. The monitoring of dog population through large serological surveys must be an important measure to indicate potential hot spots for AT, reinforcing the prevention for humans in these areas.

Veterinarians must be vigilant about the possibility of AT in dogs, especially those presenting cardiomyopathy. Despite presenting a different pathogenesis, it is crucial to perform a differential diagnosis with heartworm disease due to the cardiac clinical signs, and canine leishmaniasis because of the possibility of cross-reactivity. The development of screening serological tools to test animals in extensive surveys is extremely necessary to identify potential risk areas of exposure, and to provide information about animals moving from endemic to non-endemic areas.

Finally, cases of AT in dogs in Brazil are distributed heterogeneously in all five regions of the country, with a slight predominance in the Northeast region. Dog owners of all endemic regions must avoid triatomine-infested areas to reduce the risk of exposure to *T. cruzi* parasites. Additionally, it is advisable to update health policies about AT prevention and control in Brazil, highlighting the potential role of domiciled and stray dogs as infection source for triatomines in endemic areas.

## Supplementary Information

Below is the link to the electronic supplementary material.

Supplementary Material 1

## Data Availability

The Supplementary File 1 and dataset source used have been deposited in the Zenodo/OSF repository under the doi number 10.5281/zenodo.17128325.
