# Supplementary material for: A critical review of American Trypanosomiasis (AT) in dogs with the current distribution of cases in Brazil and a diagnostic approach for veterinary clinicians
Source: Vet Res Commun. 2026 Jan 24;50(2):134. doi: 10.1007/s11259-025-11047-6 (PMC12831677; doi:10.1007/s11259-025-11047-6)
Supplement: Supplementary file 1 — Supplementary Material 1 [file 11259_2025_11047_MOESM1_ESM.docx]

| **Region** | **State** | **Municipality (positivity)** | **Method of diagnosis** | **Overall Prevalence** | **Reference** |
| --- | --- | --- | --- | --- | --- |
| North | Tocantins | Augustinópolis (69%; 25/36), Esperantina (24%; 6/25), Axixá do Tocantins (22%; 7/32) | IFAT, ELISA, Blood culture, Blood microscopic | 37.1% (241/649) | Xavier et al. (2012) |
|  | Pará | Abaetetuba (28%; 55/197), Belém (23%; 8/34), Cachoeira do Arari (25%; 3/12), Curralinho (89%; 8/9), Monte Alegre (48%; 37/77) | IFAT, ELISA, Blood culture, Blood microscopic |  |  |
|  | Tocantins | Araguaína | IFAT, ELISA, TESA-blot | 4.50% (5/111) | Morais et al. (2013) |
|  | Pará | Abaetetuba | IFAT, Hemoculture | 26.51% (48/181) | Roque et al. (2013) |
|  | Rondônia | Monte Negro | IFAT, ELISA, Blood microscopy | 14.3% (1/7) | Bilheiro et al. (2019) |
|  | Acre | Rio Branco (8.82%; 3/34), Feijó (33.3%; 5/15), Rodrigues Alves (0%) | IFAT, ELISA | 8.98% (8/89) | Malavazi et al. (2020) |
|  | Amazonas | Manaus | PCR | Case Report | Vasconcelos et al. (2021) |
|  | Pará | Castanhal | nested-PCR | 38.9% (42/108) | Pereira et al. (2021) |
|  | Acre | Feijó (14 positive), Marechal Thaumaturgo (2 positive), Rodrigues Alves (2 positive) | IFAT, ELISA | 52.5% (133/253) | Rodrigues et al. (2022) |
|  | Pará | Abaetetuba (1 positive), Belém (1 positive), Cesárea (4 positive), Curralinho (2 positive), Ilha do Combú (11 positive), Monte Alegre (4 positive), São Domingos do Capim (2 positive) | IFAT, ELISA |  |  |
|  | Tocantins | Ananás (1 positive), Araguatins (8 positive), Nazaré (1 positive) | IFAT, ELISA |  |  |
| Northeast | Ceará | Russas | Xenodiagnosis | 8.33% (1/12) | Alencar et al. (1977) |
|  | Piauí | João Costa, Coronel José Dias, São Raimundo Nonato (Not Available) | IFAT | 11.54% (6/52) | Herrera et al. (2005) |
|  | Ceará | Jaguaruana | IFAT, ELISA | 21.88% (21/96) | Lima et al. (2012) |
|  | Paraíba | Patos | IFAT, ELISA, PCR, Blood culture, Blood microscopy | 100% (10/10) | Santana et al. (2012) |
|  | Rio Grande do Norte | Caicó | IFAT, ELISA, PCR, Blood culture, Blood microscopy |  |  |
|  | Ceará | Jaguaruana (71%; 78/110), Redenção (19%; 3/16), Russas (50%; 5/10) | IFAT, ELISA, Blood culture, Blood microscopic | 37.1% (241/649) | Xavier et al. (2012) |
|  | Piauí | João Costa (11%; 6/52) | IFAT, ELISA, Blood culture, Blood microscopic |  |  |
|  | Bahia | Buerarema | PCR | 0.74% (2/272) | Leça Júnior et al. (2013) |
|  | Paraíba | Patos | IFAT, ELISA, Indirect Hemagglutination | 4.09% (15/367) | Mendes et al. (2013) |
|  | Ceará | Tauá | IFAT, ELISA, PCR multiplex | 3.77% (20/53) | Bezerra et al. (2014) |
|  | Piauí | São João do Piauí | IFAT, ELISA | 51.94% (67/129) | Perez et al. (2016) |
|  | Paraíba | João Pessoa | IFAT, ELISA | 1.5% (6/384) | Brasil et al. (2018) |
|  | Paraíba | Brejo do Cruz | IFAT | 7.5% (15/200) | Fernandes et al. (2018) |
|  | Bahia | Ituberá | PCR | 0.51% (2/392) | Souza et al. (2018) |
|  | Rio Grande do Norte | Acari, Marcelino Vieira, Caraúbas (Not Available) | ELISA, PCR | 40% (16/40) | Araujo Neto et al. (2019) |
|  | Sergipe | Aracaju | PCR | 10.12% (17/168) | Cruz et al. (2020) |
|  | Bahia | Barra | PCR | Case Report | Silva et al. (2020) |
|  | Maranhão | São Luís | IFAT, ELISA, PCR | 17.5% (58/330) | Costa et al. (2021) |
|  | Bahia | Gentio do Ouro | ELISA, PCR | 17.5% (7/40) | Santos et al. (2021) |
|  | Ceará | Carnaubal (2 positive), Croatá (4 positive), Ibiapina (12 positive), Jaguaruana (2 positive), Redenção (1 positive), Viçosa do Ceará (4 positive) | IFAT, ELISA | 52.5% (133/253) | Rodrigues et al. (2022) |
|  | Pernambuco | Ibimirim (1 positive) | IFAT, ELISA |  |  |
|  | Piauí | São Raimundo (0 positive) | IFAT, ELISA |  |  |
| Center-West | Mato Grosso do Sul | Jaraguari | IFAT, ELISA, TESA-blot | 100% (4/4) | Souza et al. (2008) |
|  | Mato Grosso do Sul | Jaraguari | IFAT, ELISA | 22.67% (17/75) | Souza et al. (2009) |
|  | Mato Grosso do Sul | Corumbá (0/39) | IFAT, ELISA, Blood culture, Blood microscopic | 37.1% (241/649) | Xavier et al. (2012) |
|  | Mato Grosso | Cuiabá | Blood microscopy | Case Report | Almeida et al. (2013) |
|  | Mato Grosso | Várzea Grande | Necropsy, Immunohistochemical | Case Report | Pimentel et al. (2016) |
|  | Goiás | Cumari (5 positive) | IFAT, ELISA | 52.5% (133/253) | Rodrigues et al. (2022) |
|  | Mato Grosso do Sul | Urucum (28 positive) | IFAT, ELISA |  |  |
| Southeast | Minas Gerais | Bambuí | Xenodignosis | Not Available | Fernandes et al. (1992) |
|  | Minas Gerais | Uberlândia, Coromandel (8.33%) | IFAT | 8.33% (1/12) | Maywald et al. (1996) |
|  | São Paulo | Botucatu | IFAT | 0.4% (3/689) | Tome et al. (2011) |
|  | São Paulo | Ibiúna | IFAT | 6.14% (35/570) | Mascolli et al. (2016) |
|  | Espírito Santo | Guarapari | IFAT | 12.73% (7/55) | Dario et al. (2017) |
|  | Espírito Santo | Alegre, Iconha (Not Available) | ELISA | 27.78% (10/36) | Pontes et al. (2022) |
|  | Espírito Santo | Guarapari (0 positive) | IFAT, ELISA | 52.5% (133/253) | Rodrigues et al. (2022) |
|  | Rio de Janeiro | Angra dos Reis, Mangaratiba, Rio de Janeiro (0 positive) | IFAT, ELISA |  |  |
|  | São Paulo | São Paulo (2 positive) | IFAT, ELISA |  |  |
| South | Rio Grande do Sul | Porto Alegre | Necropsy, TESA-blot | Case Report | Pavarini et al. (2009) |
|  | Rio Grande do Sul | São Borja (2 positive) | IFAT, ELISA | 52.5% (133/253) | Rodrigues et al. (2022) |

**Supplementary file 1.** Distribution of *T. cruzi* canine cases across the Brazil.
